# Preliminary Favorable Outcome for Medically and Surgically Managed Extensively Drug-Resistant Tuberculosis, France, 2009–2014

**DOI:** 10.3201/eid2203.151130

**Published:** 2016-03

**Authors:** Benoît Henry, Matthieu Revest, Nathalie Dournon, Loïc Epelboin, Guillaume Mellon, Guillaume Bellaud, Pierre Mordant, Damien Le Dû, Nicolas Véziris, Christine Bernard, Sébastien Morel, Stéphane Jauréguiberry, Christian Michelet, François Bricaire, Pierre Tattevin, Éric Caumes

**Affiliations:** Centre Hospitalier Universitaire Pitié Salpêtrière, Paris, France (B. Henry, N. Dournon, L. Epelboin, G. Mellon, G. Bellaud, N. Véziris, C. Bernard, S. Morel, S. Jauréguiberry, F. Bricaire, E. Caumes);; Université Pierre et Marie Curie, Paris (B. Henry, L. Epelboin, N. Véziris, S. Morel, F. Bricaire, E. Caumes);; Centre Hospitalier Universitaire Pontchaillou, Rennes, France (M. Revest, C. Michelet, P. Tattevin);; Hôpital Européen Georges Pompidou, Paris (P. Mordant);; Université Paris Descartes, Paris (P. Mordant);; Centre Hospitalier de Bligny, Briis-sous-Forges, France (D. Le Dû);; Centre National de Référence des Mycobactéries et de la Résistance des Mycobactéries aux Antituberculeux, Paris (N. Véziris, C. Bernard);; Centre d'Immunologie et des Maladies Infectieuses, Paris (N. Véziris, S. Jauréguiberry);; Université Rennes 1, Rennes (C. Michelet, P. Tattevin)

**Keywords:** extensively drug-resistant tuberculosis, tuberculosis, TB, XDR TB, multidrug-resistant tuberculosis, MDR TB, antimicrobial resistance, bacteria, tuberculosis and other mycobacteria, outcome, bedaquiline, thoracic surgery, France

## Abstract

We report 20 cases of extensively drug-resistant tuberculosis managed in France. Treatment was individualized and included bedaquiline and linezolid for most patients and surgery in 8 patients. At last follow-up (22 months), 19 patients had achieved conversion from positive to negative on culture testing. These promising results of comprehensive management obtained in a small series deserve confirmation.

Multidrug-resistant tuberculosis (TB) and extensively drug-resistant TB (XDR TB) are among the most difficult infections to treat and are major public health concerns worldwide (*1*). In France, the number of imported XDR TB cases has dramatically increase recently, especially cases originating from countries of the former Union of Soviet Socialist Republics (*2*).

## The Study

During 2009–2014, we identified 20 persons who were admitted to 2 tertiary-care hospitals in Rennes and Paris, France, with culture-positive XDR TB infections. Patients were identified through hospital database searches; patient data were extracted from medical charts. For each patient, we performed direct examination of sputum smears; cultures on Lowenstein-Jensen medium; genotypic resistance profiling (GenoType MTBDR Plus; HAIN Lifescience, Nehren, Germany); and in vitro drug susceptibility testing (DST) on Lowenstein-Jensen medium, according to the proportions method.

All patients were isolated in negative-pressure rooms until their respiratory sample culture results converted from positive to negative (hereafter referred to as culture conversion). Medical and surgical therapeutic options were determined during multidisciplinary meetings involving infectious diseases, respiratory diseases, microbiology, and thoracic surgery departments. In agreement with World Health Organization guidelines (*3*), we selected anti-TB drug therapy on the basis of results from previously used agents or genotypic and phenotypic DST. Throughout hospitalization, treatment toxicity was carefully monitored through clinical assessments, routine laboratory tests, therapeutic drug monitoring, audiograms for patients on aminoglycosides, and weekly electrocardiograms. Treatment efficacy was monitored through thoracic imaging and monthly examination of respiratory samples. For patients with nondisseminated pulmonary TB, surgery was considered at the initiation of medical treatment if success of the treatment was deemed unlikely because of extensive lesions or after 3 months of optimized medical treatment if sputum conversion was not achieved. In agreement with procedures implemented by the French Information Protection Commission, all data were anonymized and collected on a standardized form.

The 20 XDR TB patients (Table 1) had recently arrived in France from Georgia (n = 17), Armenia (n = 2), and the Russian Federation (n = 1); median duration between arrival and hospitalization was 2 (interquartile range [IQR] 1–7) days. Median delay from admission to initiation of any anti-TB treatment was 18 (IQR 11–25) days.

**Table 1:** Clinical and demographic characteristics of 20 persons with extensively drug-resistant TB, France, 2009–2014*

Characteristic	Value†
Age, y (range)	37.1 (32–40.1)
Sex, no. patients	
M	18
F	2
Country of origin, no. patients	
Georgia	17
Armenia	2
Russian Federation	1
Body mass index, kg/m^2^ (range)	19.8 (17.7–22.7)
Past imprisonment, no. patients/no. total (%)	4/18 (22.2)
Past or present intravenous drug use, no. patients/no. total (%)	10/20 (50)
Previous history of TB, no. patients/no. total (%)	19/20 (95)
Previous anti-TB treatment, no. patients/no. total (%)	19/20 (95)
Previous thoracic surgery for TB, no. patients/no. total (%)	3/20 (15)
HIV infection, no. patients/no. total (%)	2/20 (10)
Hepatitis C virus infection, no. patients/no. total (%)	12/20 (60)
Duration of TB symptoms before current admission, y (range)	2.4 (0.5–7)
Organs involved, no. patients	
Lungs	20
Epididymis	1
Weight loss, no. patients/no. total (%)‡	15/17 (88.2)
Prolonged fever, no. patients/no. total (%)§	8/16 (50)
Hemoptysis, no. patients/no. total (%)	13/16 (81)
Serum albumin, g/L (range)	30 (26.5–32.7)
Cavitary lesions on chest radiographs or CT scan images, no. patients/no. total (%)	19/19 (100)
Multilobar radiological involvement, no. patients/no. total (%)	18/19 (94.7)

*CT, computed tomography; TB, tuberculosis. †Quantitative data are median (interquartile range); qualitative data are no. patients/no. with data available (%). ‡Loss of >5% of total bodyweight. §Body temperature >38°C during at least 3 weeks.

During the intensive phase of treatment, each patient was given 4–9 presumably active anti-TB agents (Table 2). All patients required long-term central venous access for administration of amikacin or carbapenems. Duration of medical treatment was individualized but continued a minimum of 12 months after culture conversion. Median duration of treatment was 24 (range 18–51) months. Median length of stay in the acute-care setting was 67 (IQR 55–124) days.

**Table 2:** Anti-TB agents used in the treatment of persons with extensively drug-resistant TB, France, 2009–2014*

Drug	No. patients previously treated with drug/no. with available data (%)	No. patients with resistant strains†/no. with available data (%)	No. study patients treated with drug/no. with available data (%)
Rifampin	12/15 (80)	20/20 (100)	0/20
Isoniazid	13/15 (86.7)	20/20 (100)	0/20
Pyrazinamide	14/15 (93.3)	15/17 (88.2)	9/20 (40)
Ethambutol	13/14 (92.9)	17/20 (85)	5/20 (25)
Streptomycin	6/14 (42.9)	19/20 (95)	0/20
Amikacin	3/14 (21.4)	10/20 (50)	12/20 (60)
Kanamycin	5/14 (35.7)	19/20 (95)	0/21
Capreomycin	9/15 (60)	16/20 (80)	2/20 (10)
Ofloxacin	4/14 (28.6)	20/20 (100)	0/20
Levofloxacin	3/12 (25)	NA	NA
Moxifloxacin	4/14 (28.6)	14/19 (73.7)	7/20 (35)
Ethionamide	9/15 (60)	17/20 (85)	5/20 (25)
Linezolid	0/14	0/20	20/20 (100)
p-aminosalicylate	14/17 (82.4)	4/20 (20)	16/20 (80)
Amoxicillin/clavulanate	3/14 (21.4)	NA	19/20 (95)
Imipenem	0/14	NA	19/20 (95)
Cycloserine	12/15 (80)	16/21 (76.2)	13/20 (65)
Clarithromycin	2/13 (15.4)	NA	0/21
Clofazimine	2/13 (15.4)	NA	9/18 (50)
Bedaquiline	0/20	NA	16/20 (80)

*N/A, no available data; TB, tuberculosis. †Determined by in vitro susceptibility testing at admission.

The following grade 3 or 4 treatment-associated adverse events (Common Terminology Criteria for Adverse Events, http://evs.nci.nih.gov/ftp1/CTCAE/CTCAE_4.03_2010-06-14_QuickReference_5x7.pdf) were reported in 18 patients: digestive side effects (14 patients), hepatitis (1 patient), peripheral neuropathy (7 patients), neuropsychiatric side effects (3 patients), and hearing impairment (3 patients). Candidemia developed in 5 patients. No grade 3 or 4 nephrotoxicity or cytopenia or significant QT interval prolongation were reported. Adverse events related to anti-TB agents were managed by a wide range of symptom treatments.

Thoracic surgery was performed in 8 patients (lobectomy in 5, pneumonectomy in 3), of whom 2 had severe postsurgical complications. Six of these patients were culture-positive before surgery, and their cultures converted a median of 25 days postsurgery (IQR 20–33 days).

After a median follow-up of 22 (IQR 15–27) months, 18 patients were alive; 2 had died of causes considered unrelated to TB. Sputum cultures had converted for 19 patients, 4 of whom had completed treatment and a median of 38 (IQR 29–40) months of posttreatment follow-up. Median time from treatment initiation to culture conversion was 100 (IQR 75–114) days. One patient is still under treatment and has not experienced culture conversion. No patients were lost to follow-up.

## Conclusions

This small series of XDR TB cases managed in a high-income country illustrates the potential safety and efficacy of multidisciplinary and individualized treatment with a multidrug regimen that includes new anti-TB agents (e.g., bedaquiline); innovative use of older agents (e.g., linezolid); and the combination of imipenem plus amoxicillin/clavulanate. The series also emphasizes difficulties faced by healthcare professionals caring for XDR TB patients.

The final outcome could not be ascertained for most study patients because they are still receiving antimycobacterial therapy; however, the 90% survival rate after a median follow-up of 22 months after treatment initiation is reassuring and compares favorably with survival rates of 66%, 54%, and 38% in the United States (*4*), South Africa (*5*), and the United Kingdom (*6*), respectively. The high rate of microbiologic conversion in our study (95%) also reflects the potentially achievable treatment efficacy, even in the context of previously treated XDR TB cases.

Our findings should be interpreted cautiously because of the small number of patients and the relatively short follow-up at the time of this writing. However, the overall figures of culture conversion are more satisfactory than those previously reported in high-income countries, where conversion rates ranged from 46.7% to 76.1% (*4*,*6*–*10*), and in high-prevalence settings, where conversion rates are lower (*5*,*11*,*12*). Preliminary results from a study conducted in South Africa were more favorable: samples from 48 (76%) of 63 patients with 6 months follow-up were culture-negative (*13*). Postsurgery sputum conversion was rapidly achieved for patients in our study; thus, pulmonary resection surgery, although risky, may also have contributed substantially to treatment successes.

Numerous difficulties were encountered during the study. Patients were referred to our centers soon after arriving in France, causing communication difficulties for patients with a limited understanding of French and English. Medical histories were long and complex, and most patients were in advanced stages of pulmonary TB.

The numerous side effects observed during prolonged anti-TB regimens must be optimally and intensively managed; otherwise, patients may not complete treatment. Because of the limited number of potentially active anti-TB agents, drugs with documented long-term toxicities must also be included in multidrug regimens. The high incidence of breakthrough candidemia cases (5/20 patients [25%]) was not anticipated, although prolonged exposure to broad-spectrum antimicrobial drugs and long-term central venous access are acknowledged risk factors for candidemia. This risk must be taken into account when considering treatment of XDR TB with carbapenems and amoxicillin/clavulanate. The overall good tolerability of linezolid may be a result of the low dosage (routinely, 600 mg/d initially, decreased to 300 mg/d if toxicity is suspected, even with limited evidence).

Financial, social, and cultural aspects of the management of vulnerable and marginalized patients are also essential and time-consuming. Prolonged hospital stays were necessary for patients in our study, resulting in high healthcare costs, as previously reported in South Africa (*14*). Limited resources and vulnerability are risk factors for noncompliance and disease progression. However, failure to adequately address these issues would translate into additional XDR TB transmission in the community and increased illness and death, which could result in a much higher societal burden. Previous experience in high-income countries has documented that comprehensive care of TB patients is cost-effective, even in the most vulnerable and marginalized populations, especially when multidrug-resistant or XDR TB are involved (*15*).

Our results reflect the situation in a high-income setting with free access to all potentially active drugs, extensive investigation of responsible strains (e.g., using DST and genotypic tests), daily monitoring of adverse events, regular multidisciplinary meetings to tailor treatment to any new event and evaluate the need for thoracic surgery in selected cases, dedicated medical and paramedical staff, and psychosocial support. Unfortunately, the situation may not be the same in the countries most affected by XDR TB.

## References

[1] Jacobson KR, Tierney DB, Jeon CY, Mitnick CD, Murray MB. Treatment outcomes among patients with extensively drug-resistant tuberculosis: systematic review and meta-analysis. Clin Infect Dis. 2010;51:6–14. 10.1086/653115 20504231 PMC4013786

[2] Bernard C, Brossier F, Sougakoff W, Veziris N, Frechet-Jachym M, Metivier N, A surge of MDR and XDR tuberculosis in France among patients born in the Former Soviet Union. Euro Surveill. 2013;18:20555 . 10.2807/1560-7917.ES2013.18.33.20555 23968874

[3] World Health Organization. Guidelines for the Programmatic Management of Drug-Resistant Tuberculosis. 2011 update [cited 2015 Apr 4]. http://www.ncbi.nlm.nih.gov/books/NBK148644/23844450

[4] Shah NS, Pratt R, Armstrong L, Robison V, Castro KG, Cegielski JP. Extensively drug-resistant tuberculosis in the United States, 1993–2007. JAMA. 2008;300:2153–60. 10.1001/jama.300.18.2153 19001626

[5] Pietersen E, Ignatius E, Streicher EM, Mastrapa B, Padanilam X, Pooran A, Long-term outcomes of patients with extensively drug-resistant tuberculosis in South Africa: a cohort study. Lancet. 2014;383:1230–9. 10.1016/S0140-6736(13)62675-6 24439237

[6] Abubakar I, Moore J, Drobniewski F, Kruijshaar M, Brown T, Yates M, Extensively drug-resistant tuberculosis in the UK: 1995 to 2007. Thorax. 2009;64:512–5. 10.1136/thx.2008.108712 19318348

[7] Banerjee R, Allen J, Westenhouse J, Oh P, Elms W, Desmond E, Extensively drug-resistant tuberculosis in California, 1993–2006. Clin Infect Dis. 2008;47:450–7. 10.1086/590009 18616396

[8] Bendayan D, Hendler A, Polansky V, Weinberger M. Outcome of hospitalized MDR-TB patients: Israel 2000–2005. Eur J Clin Microbiol Infect Dis. 2011;30:375–9. 10.1007/s10096-010-1096-7 20972692

[9] Minion J, Gallant V, Wolfe J, Jamieson F, Long R. Multidrug and extensively drug-resistant tuberculosis in Canada 1997–2008: demographic and disease characteristics. PLoS ONE. 2013;8:e53466. 10.1371/journal.pone.0053466 23326436 PMC3541271

[10] Jeon DS, Kim DH, Kang HS, Hwang SH, Min JH, Kim JH, Survival and predictors of outcomes in non-HIV–infected patients with extensively drug-resistant tuberculosis. Int J Tuberc Lung Dis. 2009;13:594–600 . 19383192

[11] Diacon AH, Pym A, Grobusch MP, de los Rios JM, Gotuzzo E, Vasilyeva I, Multidrug-resistant tuberculosis and culture conversion with bedaquiline. N Engl J Med. 2014;371:723–32. 10.1056/NEJMoa1313865 25140958

[12] Shean K, Streicher E, Pieterson E, Symons G, van Zyl Smit R, Theron G, Drug-associated adverse events and their relationship with outcomes in patients receiving treatment for extensively drug-resistant tuberculosis in South Africa. PLoS ONE. 2013;8:e63057. 10.1371/journal.pone.0063057 23667572 PMC3646906

[13] Ndjeka N, Conradie F, Schnippel K, Hughes J, Bantubani N, Ferreira H, Treatment of drug-resistant tuberculosis with bedaquiline in a high HIV prevalence setting: an interim cohort analysis. Int J Tuberc Lung Dis. 2015;19:979–85. 10.5588/ijtld.14.0944 26162365

[14] Pooran A, Pieterson E, Davids M, Theron G, Dheda K. What is the cost of diagnosis and management of drug resistant tuberculosis in South Africa? PLoS ONE. 2013;8:e54587. 10.1371/journal.pone.0054587 23349933 PMC3548831

[15] Yong Kim J, Shakow A, Mate K, Vanderwarker C, Gupta R, Farmer P. Limited good and limited vision: multidrug-resistant tuberculosis and global health policy. Soc Sci Med. 2005;61:847–59. 10.1016/j.socscimed.2004.08.046 15896895

